# Cyclin D1 Expression and Its Correlation with Histopathological Differentiation in Oral Squamous Cell Carcinoma

**DOI:** 10.1100/2012/978327

**Published:** 2012-05-02

**Authors:** Swati Saawarn, Madhusudan Astekar, Nisheeth Saawarn, Nidhi Dhakar, Shitalkumar Gomateshwar Sagari

**Affiliations:** ^1^Department of Oral Pathology and Microbiology, Peoples Dental Academy, Bhanpur, Bhopal 462037, MP, India; ^2^Department of Oral Pathology and Microbiology, Pacific Dental College and Hospital, Udaipur 313024, India; ^3^Department of Oral Medicine and Radiology, Peoples College of Dental Sciences, Bhopal 462037, India; ^4^Department of Oral Pathology and Microbiology, Darshan Dental College and Hospital, Udaipur 313011, India; ^5^Deprtment of Oral Pathology and Microbiology, Jodhpur Dental College and Hospital, Jodhpur 342001, India

## Abstract

*Background*. Cyclin D1 regulates the G1 to S transition of cell cycle. Its deregulation or overexpression may lead to disturbance in the normal cell cycle control and tumour formation. Overexpression of cyclin D1 has been reported in various tumors of diverse histogenesis. This case control retrospective study was carried out to study the immunohistochemical reactivity and expression of cyclin D1 and its association with site, clinical staging, and histopathological differentiation of oral squamous cell carcinoma (OSCC). *Methods*. Forty formalin-fixed paraffin-embedded tissue blocks of biopsy specimens of oral squamous cell carcinoma were immunohistochemically evaluated for expression of cyclin D1. *Results*. Cyclin D1 expression was seen in 45% cases of OSCC. It did not correlate with site and clinical staging. Highest expression was seen in well-differentiated, followed by moderately differentiated, and poorly differentiated squamous cell carcinomas, with a statistically significant correlation. *Conclusion*. Cyclin D1 expression significantly increases with increase in differentiation.

## 1. Introduction

The multistage process of carcinogenesis involves the progressive acquisition of mutations and epigenetic abnormalities in the expression of multiple genes, with an important group among them being those involved in cell cycle control [1].

The orderly progression of the cells through the various phases of cell cycle, namely, G1, S, G2, and M phases is precisely governed by a series of proteins called “cyclins,” which exert their effect by binding and activating the cyclin-dependent kinases (CDK) [2].

Cyclin D1, a 45 kD (kilo Dalton) protein encoded by cyclin D1 gene (CCND1) located on chromosome 11q13, is a part of the molecular system that regulates the cell cycle G1 to S transition [2]. It was first isolated as Parathyroid adenomatosis 1 gene (PRAD1) oncogene clonally rearranged and overexpressed in parathyroid adenomas and is identical to B-cell lymphoma1 gene (bcl-1) protoncogene, which is translocated and overexpressed in a subset of B-cell neoplasms [1].

Overexpression of cyclin D1 leads to shortening of G1 phase and less dependency on growth factors resulting in abnormal cell proliferation which in turn might favour the occurrence of additional genetic lesions [1].

Cyclin D1 expression has been studied in various carcinomas including oral squamous cell carcinomas. Some studies have been carried out to correlate the expression of cyclin D1 with histological grading of this neoplasm [2–5]. However, the results have not been consistent and conclusive.

Hence, this retrospective laboratory-based study was undertaken to study the expression of cyclin D1 in oral squamous cell carcinoma and to correlate its expression with histological differentiation.

## 2. Materials and Methods

Forty formalin-fixed paraffin-embedded (FFPE) tissue blocks of incisional biopsy specimens, which were histologically diagnosed as oral squamous cell carcinoma (OSCC), were retrieved from the archives of Department of Oral and Maxillofacial Pathology of the Pacific Dental College, Udaipur, India. One FFPE tissue block of normal oral mucosa was included as a positive control and one squamous cell carcinoma tissue with exclusion of primary antibody was used as negative control.

From each FFPE tissue block, 3-4 *μ* thick sections were cut and stained by H&E stain for histopathological grading. Tumours were graded according to Broder's criteria [6] into well- (WDSCC; *n* = 13), moderately (MDSCC; *n* = 16), and poorly differentiated (PDSCC; *n* = 11) ones.

Immunohistochemical study was carried out using polymer-labelling technique (Dako, Envision). Sections were dewaxed, washed in alcohol and antigen retrieval was carried out in a Decloaking Chamber (Pascal) with 10 mM Citra solution at 125°C for 30 seconds followed by 90°C for 10 seconds. Slides were cooled naturally and brought to room temperature. Slides were placed inside the Dako Autostainer Universal Staining System, an automated immunohistochemistry staining system. Endogenous peroxidase was blocked by using 0.3% hydrogen peroxide in methanol at room temperature for 10 minutes. Slides were washed with phosphate-buffered saline (PBS) briefly and incubated with primary antibody (Cyclin D1) for 60 minutes. Section were again washed with PBS, incubated with the polymer for 30 minutes, and washed again with PBS. Diaminobenzidine (DAB) was used as the chromogen in hydrogen peroxide for 10 minutes. Sections were then counterstained with haemotoxylin, mounted, and studied under light microscope for immunorecativity.

**Figure 1:**
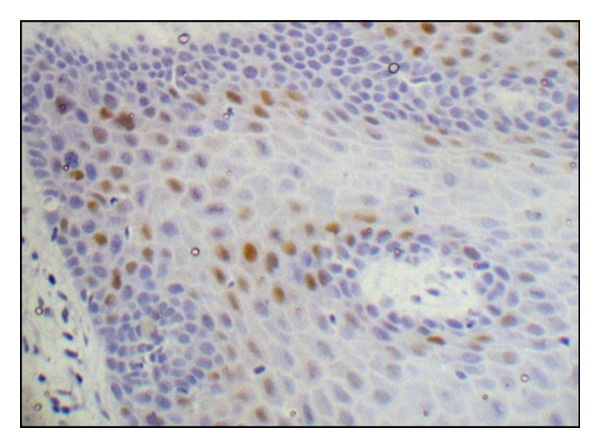
Photomicrograph depicting Cyclin D1 expression in basal and suprabasal layers of normal epithelium.

Presence of brown-coloured end product at the site of target antigen was indicative of positive immunoreactivity. The negative control demonstrated the absence of staining. Tissue section of positive control showed brown staining of the cells of basal and parabasal layers and was confirmed as being positively stained (Figure 1). The evaluation of study cases was done subsequently in a similar way for IHC reactivity. Only the slides showing positive reactivity were further evaluated for cyclin D1 expression as per criteria described by Gu et al. [7].

**Figure 2:**
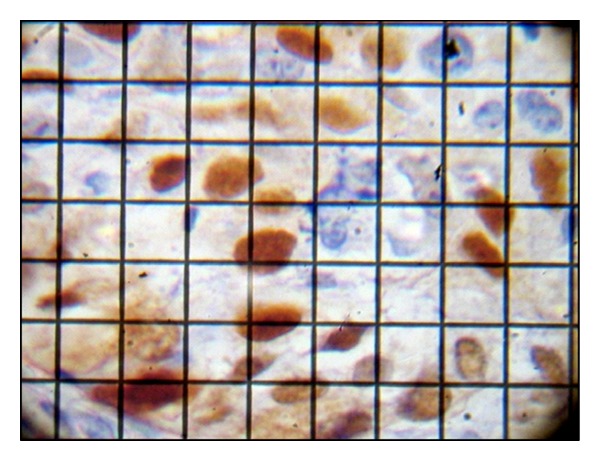
Photomicrograph depicting labelling index calculation under high magnification with a grid.

In every slide ten, hot spot areas were selected and observed under higher (400X) magnification with a grid. Percentage of IHC positive tumour cells per hot spot was calculated and the mean percentage per slide (labelling index) was determined (Figure 2). A labelling index score of 1, 2, 3, or 4 was assigned for labelling indices 1–25%, 26–50%, 51–75%, and >75%, respectively.

**Figure 3:**
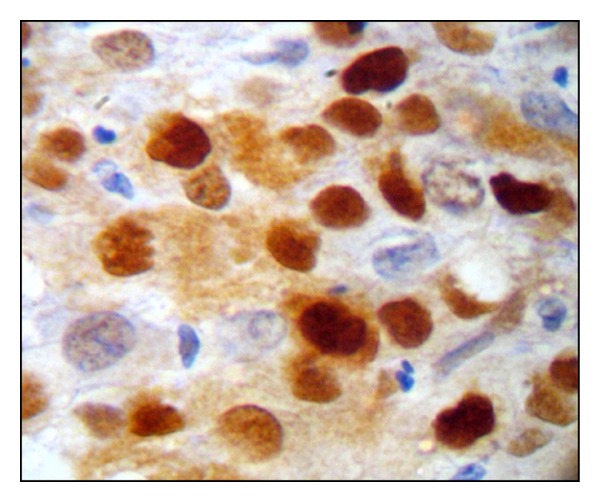
Photomicrograph depicting intensity evaluation under high magnification.

The intensity of cyclin D1 immunostaining (Figure 3) was evaluated on the basis of microscopic appearance as weak, intermediate, or strong and an intensity score of 1, 2, or 3 was assigned to them, respectively.

A final expression score was calculated by multiplying labelling index score with intensity score, based on which the cyclin D1 expression was determined as weak (score 1–4), moderate (score 5–8), or strong (score 9–12).

All the relevant clinical, histopatholocal, and immunohistochemical data so obtained were tabulated and subjected to appropriate statistical analysis using the SPSS 11 statistical software.

## 3. Results

Cyclin D1 positivity was seen in 18 cases (45%) of OSCC. Further distribution of cyclin D1 reactivity in accordance with site, clinical stage, and histopathological differentiation are explained in Table 1. The labelling index scores, intensity of staining, expressions graded, and their correlations with clinical and histological parameters are elaborated in Tables 2 and 3.

Both cyclin D1 reactivity and expression did not show any correlation with site and clinical staging of the OSCC (Tables 1 and 3). The histopathological differentiation showed a positive correlation with increase in both the reactivity and expression with increasing differentiation (Tables 1 and 3). The labelling index score and intensity did not correlate with OSCC differentiation (Table 2). 

## 4. Discussion

The present study was carried out to study the immunohistochemical reactivity and expression of cyclin D1 and its association with site, clinical staging, and histopathological differentiation of oral squamous cell carcinoma.

The IHC reactivity for cyclin D1 was evaluated on the basis of presence or absence of brown staining. Nuclear and cytoplasmic stainings in all the cases of positivity were observed, which was similar to other studies [3, 4, 7–19]. However, Gillett et al. [20] considered only cytoplasmic staining as negative, and Vora et al. [5] reported exclusively cytoplasmic staining in their cases and considered the same as positive. 

De Falco et al. have stated that in adult tissues, cyclin D1 plays a role in proliferation and differentiation and the shift between nucleus and cytoplasm is necessary to regulate finely the passage across different phases of the cell cycle. The immunogold observations have indicated transit of cyclin D1 between nuclear and cytoplasmic compartments via nuclear pores [17].

In the present study, cyclin D1 reactivity was seen in 45% of cases. Some authors have reported less than 45% reactivity [4, 8, 9, 13–16, 21] while some have shown more [2, 3, 5, 10, 12, 16, 22–25]. These reported variations in reactivity may be due to diverse reasons like asymmetric labelling expression seen in different parts of same specimen owing to the fact that in a specimen at a given time, only about 20% of the neoplastic cells are under mitosis [8]. A discrepancy in staining between the biopsy and surgical resection materials has also been reported, most likely owing to tissue heterogeneity [26]. Further, cyclin D1 has been described to express itself mainly in the peripheral layers of tumour islands and not in the cells exhibiting mitosis [3]. The pRb gene also appears to regulate transcription of the cyclin D1 which is destabilized in pRb-negative cells and hence cells negative for pRb staining do not express cyclin D1; this may be due to the fact that sometimes cyclin D1 acts as a negative regulator of cell cycle progression [20]. Bartkova et al. reported that in several common solid tumours, cyclin D1 protein may be essential for G1 phase progression while some may have lost thier requirement [27]. Further, an inverse relationship of HPV status has also been reported with cyclin D1 expression in OSCC [18]. 

To determine the cyclin D1 expression, we used a more objective and inclusive method, as depicted by Gu et al. [7] by multiplying the intensity score with labelling index score. Whereas a few authors have used only a subjective evaluation of intensity at three point criteria as “weak, moderate, and strong” [2, 4, 28] or two point criteria as “weak and strong” [21, 29] and considered it as final expression. While some other investigators have used labelling index score only as final expression [3, 5, 9, 10, 12, 14–16, 21, 23, 24, 30]. Maahs et al. used stereologic method to determine labelling index [8].

In our study, the labelling index did not have any correlation with histopathological differentiation and the results could not be compared directly with other reported literatures, because of different criteria used for determining scores by different authors [3, 5, 9, 10, 12, 14–16, 21, 23, 24, 30].

The staining intensity was nonuniform, showing maximum cases of intermediate staining followed by weak and strong. Angadi and Krishnapillai [2] and Mishra and Das [4] noted a uniformly increasing intensity in relation to the histopathological differentiation, whereas Castle et al. [28] found no correlation.

In the present study, the cyclin D1 expression shown by maximum number of cases was weak, followed by strong, and intermediate. The expression had a significant correlation with histological differentiation. There was an increase in cyclin D1 expression with increasing differentiation, that is, highest expression was seen in WDSCC, followed by MDSCC, and PDSCC. Since no studies on OSCC are available in the literature using the similar criteria for determining expression as used in the present study, we cannot directly compare this data with that of others.

Many studies have been done on cyclin D1 in OSCC, and even though the controversy exists in the scientific literature, it opens a window of opportunity for further discussion and research in different tumours with additional different criteria like lymph node involvement and metastasis. The interpretation of the above study is precluded by its limited sample size and therefore the study should be followed further with large sample size to validate our finding. The observations in this field may contribute significantly to the patient well-being and decreased morbidity and mortality by establishing cyclin D1 as a better prognostic marker.

## Figures and Tables

**Table 1:** Distribution of site, clinical staging, histopathological differentiation of OSCC, and their IHC reactivity.

Distribution category	Total number	IHC reactivity			
		Positive (*n*)	Negative (*n*)	% of positive reactivity	*P*
Site					0.282 NS
Alveolus	11	07	04	63.63	
Buccal mucosa	13	05	08	38.46	
Tongue	15	05	10	33.33	
Lip	01	01	00	100	

Clinical staging					0.867 NS
Stage I	06	03	03	50	
Stage II	07	04	03	57.14	
Stage III	13	05	08	38.4	
Stage IV	14	06	08	42.8	

Histopathological differentiation					
WDSCC	13	09	04	69.23	0.011 S
MDSCC	16	08	08	50	
PDSCC	11	01	10	9.09	

OSCC (total)	40	18	22	45	

**Table 2:** Labelling index score, and intensity in relation to histopathological differentiation.

OSCC differentiation	Intensity				Labelling index score				
	Weak (*n*)	Intermediate (*n*)	Strong (*n*)	*P*	Score 1	Score 2	Score 3	Score 4	*P*
WDSCC	4	3	2	0.075	1	5	3	0	0.361
MDSCC	2	4	2	(NS)	1	1	6	0	(NS)
PDSCC	0	0	1		0	0	0	1	

OSCC	6	7	5		2	6	9	1	

**Table 3:** Cyclin D1 expression in relation to site, clinical staging, and histopathological distribution.

Distribution category	Cyclin D1 expression			
	Weak (*n*)	Moderate (*n*)	Strong (*n*)	*P*
Site				0.255 NS
Alveolus	04	01	02	
Buccal mucosa	01	02	02	
Tongue	04	00	01	
Lip	00	01	00	

Clinical Staging				0.866 NS
Stage I	01	01	01	
Stage II	03	01	00	
Stage III	02	01	02	
Stage IV	03	01	02	

Histopathological differentiation				
WDSCC	07	00	02	0.042 S
MDSCC	02	04	02	
PDSCC	00	00	01	

OSCC (total)	09	04	05	
